# Roast-Driven Coffee Proteome Changes Characterized by Bradford Assay, SDS-PAGE, and LC-MS

**DOI:** 10.3390/foods15030538

**Published:** 2026-02-03

**Authors:** Weiying Lu, Yumei Chen, Yuge Niu, Liangli (Lucy) Yu

**Affiliations:** 1 Institute of Food and Nutraceutical Science, Department of Food Science and Technology, School of Agriculture and Biology, Shanghai Jiao Tong University, Shanghai 200240, China; 2 Department of Nutrition and Food Science, University of Maryland, College Park, MD 20742, USA

**Keywords:** coffee proteomics, roasting, liquid chromatography–mass spectrometry, chemometrics

## Abstract

Coffee proteins are key precursors of roasting flavor. However, heat-driven changes in the bean proteome remain underexplored. This work aimed to investigate these changes and study proteomic markers of the coffee bean. The green and roasted coffee beans were quantified for their total soluble protein and compared by sodium dodecyl sulfate–polyacrylamide gel electrophoresis (SDS–PAGE) and liquid chromatography–mass spectrometry (LC–MS) proteomics. The protein profiles identified by LC–MS were processed using principal component analysis (PCA) and partial least-squares discriminant analysis (PLS–DA) modeling to identify possible roast-sensitive protein markers. The alkaline-aided aqueous extract protein concentration was reduced from 14–23 g to 3–10 g/100 g dry weight (DW). SDS–PAGE showed dominant 17–26, 34–43, and 55–72 kDa bands weakened after roasting, while high molecular peaks (>180 kDa) were present only in roasted samples. In-solution tryptic digestion yielded nine protein groups. PCA scores revealed partial separation of green and roasted groups, while PLS-DA delivered unambiguous classification (Q^2^ > 0.90 by cross-validation). The variable importance in projection scores highlighted that structural proteins in common plant beans are markedly down-regulated after roasting, indicating heat-induced structural disruption. The identified protein groups represent candidate markers associated with severe thermal treatment and provide possible molecular targets for investigating flavor precursor development.

## 1. Introduction

Coffee is one of the most widely consumed beverages worldwide [1] and constitutes a pivotal commodity in global trade [2,3]. Because of its outstanding commercial attributes, coffee has been successfully integrated into peripheral markets. Coffee by-products are also increasingly exploited as sustainable substitutes for plant-derived extracts, such as in the cosmetic and pharmaceutical sectors [4,5]. According to the International Coffee Organization (ICO), global coffee consumption reached 10.5 million tons in the 2024/2025 crop year. The two dominant commercial species, *Coffea arabica* and *Coffea canephora*, accounted for 6.1 and 4.5 million tons of worldwide exports in 2025, respectively [6].

In our previous research, chlorogenic acids (CGAs) have been established as critical markers for defining coffee quality and bioactivity [7]. We utilized ultra-performance liquid chromatography–mass spectrometry (UPLC–MS) coupled with chemometric analysis to profile and classify black instant coffee and coffee bean extracts based on their CGA signatures. However, while phenolic compounds such as CGAs provide valuable insights, coffee flavor is also profoundly influenced by proteins, which serve as key precursors for flavor formation during roasting. The Maillard reaction, pyrolysis, and other thermal processes transform proteins and amino acids into volatile aroma compounds and melanoidins. These products define the sensory character of roasted coffee. Compared with direct volatile or melanoidin markers, peptide markers produced by the enzymatic digestion are non-volatile and remain relatively stable during sample transport and long-term storage. Additionally, detection by liquid chromatography–mass spectrometry (LC-MS) may provide a more sensitive and reproducible means of detecting roasting-induced changes. Despite these recognized roles and characteristics, the coffee-bean proteome remains remarkably under-investigated. LC-MS enables confident identification and quantification of peptide sequences [8], offering an unprecedented opportunity to explore the coffee proteome in its native and processed states.

Green coffee beans contain approximately 8.7–12.2% (*w*/*w*%, dry basis) protein [9], in contrast, spent coffee grounds and the silver skin, two main roasting by-products, contain 13.5–19.5 g/100 g DW [10] and 16.2–19 g/100 g DW [11], respectively. Although coffee proteins are largely insoluble and therefore minimally bioavailable to the consumer, they are fundamental precursors of aroma-active compounds. During high-temperature roasting, free amino acids, peptides, and proteins participate extensively in Maillard chemistry, thereby dictating the final flavor and cup quality. Compared to other plant sources, coffee proteins have a superior essential amino acid profile, notably high levels of leucine, isoleucine, and valine [12]. This profile further enhances the commercial value of coffee products. Structural characterization has revealed that roasting provokes substantial modification of the coffee protein fraction. For example, Montavon et al. [9] demonstrated that the 11 S storage protein subunit becomes covalently incorporated into melanoidin structures during roasting. More recently, Böhm et al. systematically tracked protein-bound Maillard reaction products (MRPs) and amino acid modifications in coffee melanoidins across a roasting series, providing direct evidence for the rapid and sequential modification of nucleophilic amino acid side chains during thermal processing [13]. Consequently, proteomic comparison of unprocessed and roasted beans not only clarifies the molecular basis of flavor formation but also provides critical insight into genotype- and origin-specific traits that govern processability and ultimate beverage quality. For the coffee industry, translating this insight into practice is of direct commercial importance. Mapping these proteomic transformations enables more precise control over the roasting process, allowing for the tailoring of sensory profiles to meet market preferences [14]. Furthermore, identifying robust protein or peptide markers associated with thermal processing provides an objective tool for quality control, authentication of roast claims, and detection of adulteration. Ultimately, this knowledge supports optimized roasting protocols, enhances product traceability, and aids in developing novel coffee-based ingredients, adding significant value across the supply chain.

As a continuation of our study, this work extends the chemometric approach to the proteomic level. We aimed to investigate heat-driven changes in the coffee bean proteome and identify candidate protein-based features associated with thermal processing. The coffee beans were roasted and compared using three profiling techniques: Firstly, the total soluble proteins were quantified and compared by the Bradford assay. Secondly, the protein content was then evaluated by sodium dodecyl sulfate–polyacrylamide gel electrophoresis (SDS-PAGE). Lastly, following enzymatic hydrolysis, characteristic peptides from coffee were analyzed by ultra-performance liquid chromatography–mass spectrometry (UPLC-MS). Subsequently, multivariate chemometric models were constructed to assess the discriminatory power of proteomic markers as a function of thermal treatment. By studying heat-driven proteomic changes from raw to processed coffee products, this work advances coffee flavor science and furnishes a robust analytical framework to support evidence-based optimization of coffee production and processing.

## 2. Materials and Methods

### 2.1. Materials and Reagents

Seventeen raw-coffee bean lots were purchased from local producers or authorized distributors in China in 2022. Each 500 g batch of coffee beans was sealed in its original bag and stored therein until analysis. The detailed sample information is compiled in Table 1, as well as the additional original sample information, including the translated complete product label and unit price provided in Table S1 in the Supplementary Materials.

Ultrapure water was generated in-house with a Milli-Q system (Millipore Laboratory, Bedford, MA, USA). LC–MS-grade acetonitrile was supplied by Merck KGaA (Darmstadt, Germany). LC-MS grade formic acid, ammonium bicarbonate, DL-dithiothreitol (DTT), iodoacetamide (IAA), and trypsin were purchased from Sigma-Aldrich (St. Louis, MO, USA). Analytical-grade ethanol and Coomassie Brilliant Blue (CBB) G250 were purchased from Shanghai Aladdin Bio-Chem Technology Corporation (Shanghai, China). Analytical-grade phosphoric acid, hydrochloric acid, sodium hydroxide, and acetic acid were obtained from Sinopharm (Beijing, China); SDS-PAGE Gel Quick Preparation Kit (30–50 gels), 4× Laemmli sample buffer, electrophoresis buffer, pre-stained Color Protein Marker (10–180 kDa), and CBB R250 staining solution were all supplied by Beyotime Biotechnology (Shanghai, China). Bovine serum albumin was acquired from Rhawn Chemical Technology Co., Ltd. (Shanghai, China). Phosphate-buffered saline (PBS) buffer was purchased from Labgic Technology Co., Ltd. (Beijing, China).

### 2.2. Sample Pretreatments

All samples were pretreated by individual roasting at 200 °C for 4 h in a ventilated oven to simulate industrial roasting conditions. The images for all unroasted and roasted coffee beans were presented in Figure S1 in the Supplementary Materials. It can be observed that the beans exhibit distinct initial colors as expected. After roasting, all samples show a uniform darkening, confirming the roasting protocol is valid. The laboratory-roasted beans were then ground to a fine powder using an IKA laboratory grinder (Staufen, Baden-Württemberg, Germany), and the particles passing through an 80-mesh sieve were collected for subsequent analyses.

### 2.3. Extraction and Quantification of Total Soluble Protein

Exactly 100 mg of each finely ground sample was suspended in 1 mL of deionized water and vortex-mixed for 30 s. They were then sonicated for 30 min to solubilize the water-soluble protein fraction using an ultrasonic bath (KunShan Hechuang Ultrasonic Technology, Shanghai, China). After centrifugation at 1000× *g* and 18 °C for 10 min, the supernatant was collected and diluted appropriately for quantification. Bradford reagent was prepared by dissolving 100 mg CBB G250 in 50 mL 95% ethanol, adding 100 mL 85% (*w*/*w*) phosphoric acid, and bringing the volume to 200 mL with deionized water. Fifty milliliters of this stock solution were further diluted with 200 mL of water prior to use. A calibration curve (0–100 µg mL^−1^) was constructed with bovine serum albumin (BSA). Aliquots of 50 µL of each standard or sample were transferred to a 96-well microplate, mixed with 200 µL of the diluted Bradford reagent, and incubated for 10 min. Microplate assays were read on an Infinite M1000 PRO microtiter plate reader (Tecan Group Ltd., Männedorf, Switzerland). Absorbance was read at 590 nm in triplicate for every standard and sample.

### 2.4. SDS-PAGE Analysis

To obtain protein-enriched material, the alkali-solubilization/acid-precipitation protocol was adopted with minor modifications, as previously established for coffee and other plant matrices [10,15]. This protocol was selected to consistently isolate and concentrate the alkali-soluble protein fraction, which contains key flavor precursors relevant to roasting chemistry. Secondly, this method can effectively remove interfering non-proteinaceous compounds (e.g., phenolics, polysaccharides) via precipitation at the protein iso-electric point, thereby yielding a purified protein pellet suitable for downstream comparative analyses by SDS-PAGE and LC-MS. The alkali-solubilization/acid-precipitation protocol was adopted with minor modifications [10,15]. One gram of each coffee bean was suspended in 10 mL of water and vortex-mixed for 30 s. The pH was raised to 10 with 1 M sodium hydroxide, followed by 30 min of sonication. After centrifugation at 4 °C and 4000× *g* for 30 min, the supernatant was recovered, adjusted to pH 4.5 with 1 M hydrochloric acid to precipitate the protein, and centrifuged again under the same conditions. The resulting pellet was lyophilized to yield a dry extract. Each lyophilized extract was dissolved in PBS buffer (1:10 *w*/*v*) to yield a 1 mg mL^−1^ protein solution. The solution was vortexed for 30 s and sonicated for 10 min. A 2 mL aliquot was mixed with 0.5 mL 4× Laemmli sample buffer and heated at 100 °C for 5 min.

SDS-PAGE was performed on a Mini-PROTEAN Tetra cell system (Bio-Rad Laboratories, Inc., Hercules, CA, USA). The 12% separating gel and 5% stacking gel were prepared according to the manufacturer’s instructions from the SDS-PAGE Gel Quick Preparation Kit. After 20–30 min of polymerization, the surface was overlaid with water, blotted dry, and overlaid with a 10-well comb, each loaded with 5 µL pre-stained marker or 10 µL sample, which was inserted and run for 30 min at 120 V. The gel was stained with CBB R250 on a rocking platform for 90 min and de-stained for 120 min in 50% water/40% ethanol/10% acetic acid (*v*/*v*/*v*). Images were recorded with a ChemiDoc XRS+ gel-imaging system (Bio-Rad Laboratories).

### 2.5. UPLC–MS Analysis

Enzymatic hydrolysis was performed as a sample pretreatment step following the previous protocol [16] with minor modifications. Specifically, 20 mg of lyophilized coffee-protein extract was dissolved in 1 mL of 100 mM ammonium bicarbonate. The disulfide bonds were reduced by adding 10 µL 1M DTT to the mixture and reacting at 50 °C for 30 min, and alkylation was carried out by adding 30 µL 1M IAA to the mixture and keeping it in the dark for 30 min. Subsequently, 20 µL trypsin (2 mg mL^−1^) was added, and the mixture was incubated at 37 °C for 4 h to promote proteolysis. The reaction was terminated by heating at 85 °C for 10 min. After centrifugation at 13,000× *g* and 4 °C for 12 min, the supernatant was filtered through a 0.45 µm membrane and transferred to LC–MS vials for analysis. Each sample was processed and tested in duplicates.

UPLC–MS analysis was performed on an ACQUITY UPLC hyphenated with a Xevo G2 quadrupole time-of-flight-mass spectrometry (QTOF–MS) instrument system (Waters Corporation, Milford, MA, USA). The QTOF–MS was operated in positive ion electrospray mode (ESI^+^). Chromatographic separation was achieved on an ACQUITY BEH C18 column (100 × 2.1 mm, 1.7 µm) and maintained at 30 °C; the autosampler was kept at 10 °C. Mobile phase A consisted of 0.1% formic acid in water (*v*/*v*), and mobile phase B was 0.1% formic acid in acetonitrile (*v*/*v*). The injection volume was 5 µL. A gradient elution program was applied at a flow rate of 0.4 mL min^−1^ (0–2.9 min, 2% B; 3.0–23.0 min, 2–40% B at 0.2 mL min^−1^; 25.0 min, 85% B at 0.4 mL min^−1^; 27.0 min, 100% B; 27.1–30.0 min, re-equilibration at 2% B). The needle was washed after each injection with 200 µL strong-wash solvent (isopropanol/acetonitrile 9:1 *v*/*v*) and 600 µL weak wash solvent (acetonitrile).

The mass spectrometer parameters were set as follows: the desolvation temperature was set to 450 °C, the source temperature was maintained at 120 °C, the capillary voltage was fixed at 3 kV, the cone voltage was adjusted to 40 V, and the spray voltage was established at 3.0 V; the cone gas flow, utilizing nitrogen (N_2_), was regulated at 50 L h^−1^, while the desolvation gas flow, also using nitrogen, was set at 600 L h^−1^; the collision gas employed was high-purity argon with a purity of 99.999%. Mass spectra were acquired across the mass-to-charge (*m*/*z*) range of 50–1800. Data were acquired in Data-Independent Acquisition (DIA) mode using the Waters MS^E^ method. The instrument alternated automatically between two scan functions: a low-energy scan at a fixed collision energy of 6 eV to record precursor ions, and a high-energy scan with a ramped collision energy from 20 to 50 eV to record all fragment ions.

### 2.6. Proteomic Data Processing

Raw UPLC–QTOF-MS datasets were imported directly into Progenesis QI (version 2.0, Waters). The peak-picking and identification processes all used default settings from Progenesis QI, except that the automatic sensitivity was set to 5. Tryptic specificity was enforced in silico. A specifically curated protein sequence database was constructed by downloading all available entries for the genus *Coffea* (Taxonomy ID 13442) from the UniProtKB/Swiss-Prot protein sequence database (https://www.uniprot.org/, accessed on 15 January 2026), release version 2025_03). To account for common laboratory contaminants, the common Repository of Adventitious Proteins (cRAP) database (ftp://thegpm.org/fasta/cRAP, accessed on 19 January 2026) was appended to the *Coffea* sequence database prior to the search. The curated database was created by searching all records of the *Coffea* organism in the UniProtKB/Swiss-Prot. This database comprises 94,704 protein sequence entries, among them 105 entries were reviewed (Swiss-Prot). When performing identifications, ambiguous identifications were found due to the over-redundancy in the prepared protein database. To address this redundancy, protein grouping was used, where the same peptides could map to multiple protein hits. The protein grouping technique clusters indistinguishable or subset proteins into a single reported group, with the protein having the greatest peptide evidence (e.g., most unique peptides or highest sequence coverage) designated as the lead identity. This approach simplifies the protein list and resolves quantification conflicts for shared peptides. All other search settings were kept at the software default, including the automatic *m*/*z* tolerance, a maximum of one allowed missed cleavage, and a maximum protein mass of 250 kDa. The identical search and processing parameters were applied to all samples to ensure comparability. The protein quantitation was performed using relative quantitation, i.e., default settings, based on the 3 most abundant, high nitrogen-containing unique peptides. The resulting protein intensity table was exported as a spreadsheet file.

### 2.7. Statistical and Chemometric Analysis

The dataset was imported into the online version of MetaboAnalyst 6.0 (https://www.metaboanalyst.ca, accessed on 10 December 2026) [17]. Technical duplicates were analyzed independently. The primary statistical hypothesis was that the proteomic profiles of raw (green) and roasted coffee beans are distinct and can be modeled to classify samples based on thermal treatment. All data were pre-processed using only autoscaling with no other steps applied. Autoscaling involves two sequential operations for each variable: (1) mean-centering (subtracting the variable mean from each value), which sets the mean of each variable to zero; and (2) unit variance scaling (dividing each mean-centered value by the variable’s standard deviation), which gives each variable equal variance (standard deviation = 1). This transformation minimizes the influence of highly abundant proteins/peptides and ensures that all features contribute equally to the multivariate models regardless of their original concentration ranges, allowing for the detection of patterns based on relative changes rather than absolute abundance. Alternative scaling methods (Pareto scaling, range scaling, or raw data without any preprocessing) were tested and yielded consistent clustering patterns in principal component analysis, confirming the robustness of the observed group separations to different preprocessing approaches. Missing values were left as zero. Subsequently, unsupervised principal component analysis (PCA) and supervised partial least-squares discriminant analysis (PLS-DA) were employed to visualize group separation. The PLS-DA model was validated using 5-fold cross-validation. Model performance was assessed using the goodness-of-fit (R^2^) and goodness-of-prediction (Q^2^) metrics. Features important for class separation were identified based on their variable importance in projection (VIP) scores from the validated PLS-DA model. A model with Q^2^ > 0.5 is considered to have good predictive power, and (R^2^ − Q^2^) < 0.3 indicates a minimal gap between model fit and prediction. A VIP score > 1.0 was used as the threshold for selecting influential proteins.

## 3. Results and Discussion

### 3.1. Considerations on Thermal Treatment Under Isothermal Conditions

The thermal treatment was conducted in a laboratory oven to impose precise, isothermal heat under static, low-agitation conditions. This setup fundamentally differs from a dynamic industrial roaster, where high-temperature air (>250 °C), rapid bean movement, and convective heat transfer enable roast development within 8–20 min. In our static system, heat transfer is limited to low-velocity air conduction, drastically slowing moisture loss and color development. Therefore, the time of thermal treatment is explored to achieve a bean color equivalent to a standard medium roast. The preliminary trials at 200 °C were conducted for 30 min, 1 h, 2 h, and 4 h for all beans, and compared visually by a Specialty Coffee Association of America (SCAA) roast color card. The result indicated that only the 4 h treatment reliably produced the target color without surface carbonization or bean fracture, as shown in Figure S1 in the Supplementary Materials, indicating that the maximum bean temperature remained below combustion thresholds. This extended time represents the kinetic compensation required to reach the desired thermal endpoint under controlled, low-heat-transfer conditions and should not be equated with the duration of an industrial roast cycle. Our approach to laboratory oven-based heating provides reproducible, uniform thermal stress for comparative proteomic analysis. Therefore, the 4 h, 200 °C condition is presented as a controlled, non-industrial, and proof-of-concept thermal model to achieve a target roast color under isothermal conditions. It is not intended to replicate industrial roasting kinetics but to provide a reproducible benchmark for comparative proteomic analysis.

### 3.2. Differential Protein Content Between Green and Roasted Beans

The water-soluble protein contents of the green coffees and their corresponding roasted counterparts were quantified by the Bradford assay. The results are summarized in Table 2. The water-soluble protein content of the green beans ranged from 14.34 to 23.33 g/100 g DW, with an average of 16.22 g/100 g DW, in contrast, the corresponding roasted samples contained only 3.51–10.42 g/100 g DW, with an average of 7.28 g/100 g DW. It should be noted that the values obtained from the Bradford assay should be interpreted as relative quantification rather than an absolute measure of protein concentration. Nevertheless, a consistent decrease was observed for all seventeen lots after roasting, indicating heat-induced protein modifications such as degradation or aggregation [18]. Elevated temperature is known to reduce both free and total amino acid pools in coffee; Cuong et al. [19] reported substantial losses of aspartic acid, threonine, serine, lysine, and arginine under typical roasting conditions. To obtain a more detailed view of the molecular changes brought about by roasting, SDS-PAGE was employed to compare the molecular-weight distribution of proteins in green and roasted beans.

### 3.3. Differential Protein Profiles by SDS-PAGE

**Figure 1:**
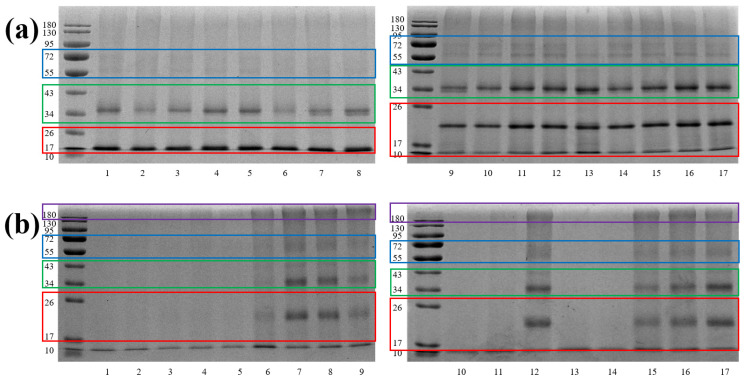
SDS-PAGE results of (**a**) raw and (**b**) roasted coffee beans. The lane numbers (1–17) correspond to the sample identifiers listed in Table 1. For better demonstration, the discussed bands have been highlighted with colored borders. Red, green, blue, and violet, respectively, indicate bands of 17–26, 34–43, 55–72, and >180 kDa.

The SDS-PAGE profiles of green and roasted beans are displayed in Figure 1. Three predominant molecular-weight regions were resolved: 17–26, 34–43, and 55–72 kDa. Bands corresponding to green beans were consistently more intense than those of their roasted counterparts, corroborating the higher water-soluble protein content determined by the Bradford assay (Section 3.2). Within the roasted set, samples 6, 7, 8, 9, 12, 15, 16, and 17 yielded noticeably stronger staining, implying superior protein retention after heat treatment. Faint high-molecular-weight smears (>180 kDa) appeared exclusively in roasted samples, indicative of heat-mediated aggregation or cross-linking. Because SDS-PAGE cannot distinguish proteolysis from aggregation or cross-linking, we refrain from describing proteomic changes as degradation or other modifications. Definitive demonstration of peptide-bond cleavage would require reducing/native PAGE comparisons combined with targeted bottom-up proteomics of TCA-soluble fractions with semi-tryptic searches, which lies outside the scope of this work. Another limitation of this study is the use of a selective protein extraction protocol (alkali solubilization/acid precipitation), which focuses on the proteins soluble at high pH and precipitable at their isoelectric point. This approach does not capture proteins that become acid-soluble or irreversibly incorporated into the insoluble melanoidin fraction during roasting, a known major pathway for coffee proteins. Future work employing sequential extraction buffers (e.g., acidic, neutral, and detergent-containing) would provide a more comprehensive view of roast-induced proteome solubility shifts. To obtain a more comprehensive view of these modifications, the identical extracts were subjected to in-solution tryptic digestion followed by UPLC-QTOF-MS analysis.

### 3.4. Proteomic Profiling

Following automatic peak alignment, exhaustive peak picking, and normalization, a consolidated peptide-feature matrix that consisted of 8849 peptides was generated. A total of 9 proteins were tentatively identified from the 34 coffee extracts by Progenesis QI (Table 3). The observed molecular mass distribution (12–130 kDa) agreed closely with the SDS-PAGE data. The resulting protein abundance data, forming a 68 × 9 (number of objects × number of variables) input matrix subjected to chemometrics analyses, are given in Table S2 in the Supplementary Materials. The observed molecular mass distribution (12–130 kDa), coupled with the identification of dominant 11S globulin storage proteins, is characteristic of a mature seed proteome. The dominant functional theme is nutrient storage and seed development, evidenced by the prevalence of 11S globulin storage proteins. These proteins are not unique to coffee, instead they are evolutionarily conserved core components of seeds across diverse plant families, including legumes (soybean, pea), nuts, and cereals. Each individual identification of groups represents different aspects of functions. Protein G1 matched 11S globulins that are widely applied in the food industry [20]. The 11S globulin has 490 amino acids and 6 heterodimeric subunits linked by disulphide bonds, corresponding to what was previously reported in coffee by Montavon et al. [9]. Proteins G2 and G9 have limited research in coffee beans [21], but they have been reported to be crucial components of seed reserves, playing a significant role in plant development and germination, such as in pea [22] and *Arabidopsis* seeds [23]. Proteins G5 and G7 were identified as Bet v 1/Major latex protein (MLP) domain-containing proteins, a diverse family of plant proteins with significant roles in plant defense mechanisms and as allergens. Proteins G6 and G8, Cupin-type-1 domain-containing proteins, also represent a diverse superfamily involved in various plant biological processes. Functional annotation for the proteins is currently limited in *Coffea*, reflecting the general paucity of relevant intact protein studies [21].

The low protein count reflects our focus on the soluble, flavor-reactive major fraction. During the 4 h static-heat treatment, the bulk of storage proteins becomes insoluble or is entrapped in melanoidins [9]. Our mild extraction, therefore, recovers only the subset available for Maillard chemistry. Standard-flow UPLC-QTOF-MS was chosen for robust quantification of these abundant peptides rather than exhaustive proteome coverage, an approach previously validated by our group [16]. Searches were restricted to the UniProtKB *Coffea* genus to minimize false positives, but this stringency—combined with the still-limited annotation of processed coffee proteins—naturally reduces identifications while ensuring each reported protein is of high confidence. While SDS-PAGE indicates protein modification that could theoretically limit tryptic access, the successful LC-MS detection of peptides across all roast levels, particularly thermostable fragments from storage proteins, confirms that our extraction protocol provided a representative digestible fraction. Therefore, the identified peptide markers reliably reflect roast-driven proteome changes despite the modified state. Furthermore, as the protein grouping that is applied here to manage complex identifications is a pragmatic simplification, the result may be obscured by specific isoforms or paralogs. As a result, the biological implications of protein hits should be interpreted with caution. The list of identified proteins was retained as candidate markers for subsequent chemometric modeling to discriminate between the raw and roasted beans.

**Table 3:** Tentatively identified proteins in coffee beans by UPLC–QTOF–MS.

ID *	Group Accessions	Peptide Counts (Unique Peptides) *	Description	Organism
G1	A0ABM4X233 P93079 Q9SAN3	136 (10)	11S globulin seed storage protein Ana o 2.0101-like	*Coffea arabica*
G2	A0ABM4V1S7 A0A068U2P4	10 (2)	Vicilin-like seed storage protein At4g36700	*Coffea arabica*
G3	A0A068VLE8 A0A6P6V351 A0A6P6VBJ4	7 (7)	DH200 = 94 genomic scaffold, scaffold_4051	*Coffea canephora*
G4	A0A068UXY8 A0A6P6UXR6 A0A6P6VAA7 G4Y630; G4Y631	10 (10)	Uncharacterized protein	*Coffea canephora*
G5	A0A068UPZ8, etc. *	24 (24)	Bet v I/Major latex protein domain-containing protein	*Coffea canephora*
G6	A0A6P6T6X1 A0ABM4V5N1	9 (1)	Vicilin-like seed storage protein At4g36700 isoform X1	*Coffea arabica*
G7	A0A068UK55 A0A6P6U7L1 A0A6P6XJ76	10 (9)	Vicilin-like seed storage protein At2g28490	*Coffea canephora*
G8	A0A068TW24 A0A6P6V4X9 A0A6P6VA69	8 (8)	Bet v I/Major latex protein domain-containing protein	*Coffea canephora*
G9	A0A068TV85 O82437 Q9ZNY2	133 (7)	Cupin-type-1 domain-containing protein	*Coffea arabica*

* ID, protein group ID in this study; peptide count is the entire set of peptide features that could be confidently matched to the protein, including both peptides that map only to that single entry and peptides whose sequence is shared with other proteins in the database; unique peptides is the subset of peptide hits that their sequence occurs exclusively in the protein group entries. The complete Accession IDs for proteins #5 are as follows: A0A068UPZ8; A0A068U7G3; A0A068UP63; A0A068UPT5; A0A068VKH2; A0A6P6U8U8; A0A6P6UAU8; A0A6P6UCV6; A0A6P6VI19; A0A6P6WNH4; A0A6P6WNI7; A0A6P6WNL6; A0A6P6WQQ5; A0A6P6WVA8; A0A6P6WXC1; and A0ABM4VCC9.

The integrated use of SDS-PAGE and LC-MS provided a multi-scale analytical framework, leveraging their complementary advantages and limitations. SDS-PAGE offered the key advantage of directly visualizing bulk protein integrity and aggregation states, which is challenging to infer from peptide-level LC-MS data. Its main disadvantage is low resolution and an inability to identify specific proteins or peptides. LC-MS provided the key advantage of high-resolution, sensitive identification and quantification of specific peptides and proteins. Its main disadvantage in this context is that it is costly, requires significant capital investment, specialized expertise, complex operation steps, and is less routinely accessible than SDS-PAGE in many quality-control settings. In practice, SDS-PAGE offered a direct, global snapshot of the coffee proteome’s fate under thermal stress, confirming the expected transition from discrete bands in green beans to a high-molecular-weight smear and eventual signal loss. This result served as essential quality control, verifying that sample preparation was effective and that the roasting protocol induced the profound proteomic changes necessary for subsequent molecular-level investigation. In contrast, LC-MS enabled specific, peptide-resolved quantification and identification. While SDS-PAGE indicated bulk protein loss, LC-MS pinpointed the specific proteins most susceptible to degradation or aggregation. These proteins constitute the most promising candidate targets for future roast authentication studies. Thus, SDS-PAGE validated the global-level profile, while LC-MS elucidated the specific molecular identities, ensuring precise biomarker discovery.

Acknowledging that the distinct analytical principles of each technique are crucial for holistic interpretation. SDS-PAGE is subject to biases, including differential staining affinity based on protein composition and the selective extraction of only SDS-soluble proteins, potentially under-representing aggregated or melanoidin-bound fractions. Conversely, the LC-MS results are influenced by inherent biases of bottom-up proteomics: peptide ionization efficiency in the electrospray source varies dramatically by sequence, and trypsin digestion efficiency can be uneven, both of which skew quantitative representation. Most fundamentally, LC-MS provides a peptide-centric view, while SDS-PAGE visualizes intact protein mobility. Therefore, the concordance between the two techniques—where the bulk protein depletion observed by SDS-PAGE aligns with the quantitative reduction in specific proteins identified by LC-MS—is not trivial. It suggests that the observed trends (e.g., the loss of specific storage proteins) are robust analytical findings, less likely to be artifacts of a single method’s bias. The SDS-PAGE validates the macromolecular consequence of roasting, while LC-MS defines the specific molecular targets, with each method compensating for the other’s blind spots.

### 3.5. Chemometric Classification

**Figure 2:**
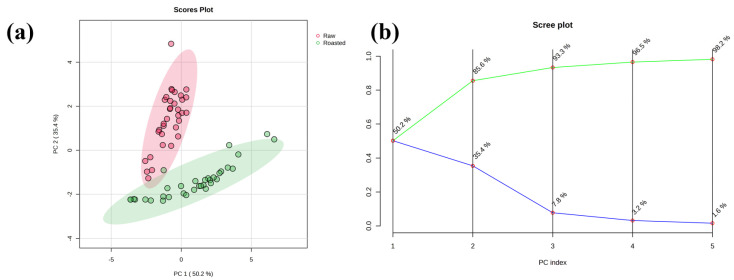
PCA scores plot (**a**) and accumulated variance explained plot (scree plot) (**b**). In the PCA scores plot, the raw and roasted samples were shown in red and green circles, respectively. The 95% confidence regions were displayed in ellipses. In the scree plot, a descending variance of the principal components was given in the blue line, and the cumulative variance was shown in the green curve.

PCA and PLS-DA models were constructed to classify green and roasted beans. The PCA score plot (Figure 2a) shows a separation between the two groups, with close but distinct region according to the 95% confidence eclipses. The first two principal components account for 85.6% of the total variance. Specifically, the scree plot analysis (Figure 2b) demonstrates a typical diminishing returns pattern in variance explanation across the principal components, with PC1 explaining 50.2% of the total variance, PC2 contributing an additional 35.4%, and PC3 contributing a further 7.8% (cumulative 93.3%). The 95% confidence regions were displayed in ellipses. In the scree plot, a descending variance of the principal components in the blue line marks the cutoff between informative components and noise, and the green cumulative curve flattens at the elbow where later PCs add negligible information and may be discarded as noise. The subsequent components show substantially reduced explanatory power. This distribution confirms the validity of retaining the first two principal components for the PCA in this study, as they capture the vast majority of meaningful variation and avoid overfitting. A labeled PCA scores plot with all technical replicates is also provided in Figure S2 in the Supplementary Materials. It can be observed that the replicates cluster closely together within their respective sample groups (raw or roasted), which reinforces the robustness of the measured proteomic profiles.

**Figure 3:**
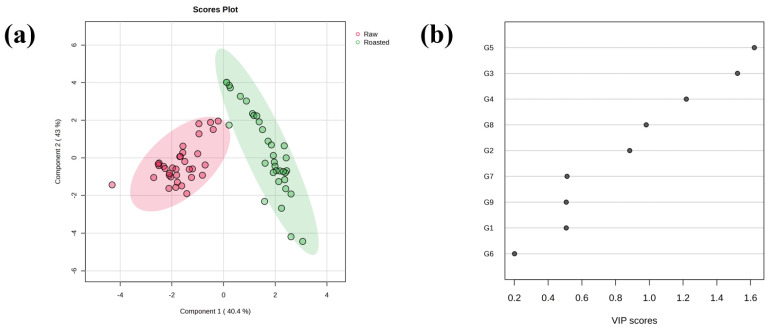
PLS-DA scores (**a**) and accumulated variance explained plots (**b**). The 95% confidence regions were displayed as ellipses. In the PLS-DA scores plot, the raw and roasted samples were shown in red and green circles, respectively. The number on the left corresponds to the protein group ID in Table 3, and the VIP scores were plotted in descending order.

Because PCA is unsupervised and does not incorporate class labels, the close separation between green and roasted bean clusters can limit its ability to define a clear classification boundary. Consequently, a supervised method such as PLS-DA becomes necessary to promote group separation and identify the specific variables that drive class distinction. To achieve better class discrimination, a PLS-DA model was further developed. Figure 3a is the PLS-DA scores plot that demonstrates unambiguous separation, with the first and second largest principal components explaining 83.4% of the overall variance. The corresponding PLS-DA scores plot with detailed sample labels is provided in Figure S3 in the Supplementary Materials. The close clustering of replicates within each class further validates the model’s stability and the consistency of the proteomic data. The corresponding VIPs (Figure 3b) reveal that four protein groups (G5, G3, G4, and G8) were with VIPs > 1, all of which are more abundant in raw beans. In particular, protein group G5 constitutes the most discriminatory features. The unambiguous separation between raw and roasted beans, attributed to the higher abundance of most protein groups in the raw state, suggests that heating leads to the degradation or structural alteration of these proteins. These findings were also consistent with a previous experiment of total soluble protein quantification, where roasting reduced the water-soluble protein content from an average of 16.2 g/100 g DW in the green beans to 7.3 g/100 g DW in the roasted counterparts. Therefore, these proteins may serve as robust markers for distinguishing green from roasted coffee and may warrant further investigation into the molecular mechanisms underlying the thermal degradation and aggregation of bean proteins.

While PCA relies on visual interpretation of score plots for pattern recognition and sample grouping, PLS-DA can be operated as a fully automated classification system. It mathematically determines sample class membership through supervised learning, other than the visual presentation of scores. Cross-validation validates this automated approach. It partitions the dataset, trains the model on subsets, and predicts classifications for held-out samples, providing an objective assessment of accuracy. As a result, the PLS-DA model was further tested with 5-fold cross-validation to evaluate the accuracy of automatic classification. This approach is performed without requiring any human intervention or manual observation of score plots, distinguishing it fundamentally from exploratory methods like PCA. The optimal model complexity of four latent variables, i.e., components, was selected based on achieving the highest Q^2^ value of 0.87461 while maintaining perfect accuracy. The model achieved 100% accuracy with an R^2^ of 0.89974. These results indicate that the PLS-DA model successfully discriminated between roasted and raw samples in the training data while demonstrating excellent predictive ability, with R^2^ showing strong model fit and Q^2^ confirming reliable cross-validation performance for distinguishing coffee types based on their protein profiles. Summing up, our results not only provide a proteomic perspective on coffee roasting but also establish a robust analytical framework for detecting thermal treatment, complementing and expanding metabolomic-based classification of plant-derived foods and ingredients.

## 4. Conclusions

Advancing the understanding of thermally induced protein alterations is therefore essential for a comprehensive appraisal of coffee quality. This study investigated heat-induced proteomic changes in coffee beans subjected to roasting. Water-soluble protein concentrations decreased 2- to 4-fold after 4 h at 200 °C. SDS-PAGE revealed a predominance of low-molecular-mass proteins (17–26, 34–43, and 55–72 kDa). Tryptic digestion coupled to UPLC–QTOF–MS retrieved nine major functional protein groups common to plant seeds, such as the key storage protein 11S globulin. When these proteins were used as chemical descriptors, PCA and PLS-DA models achieved unambiguous separation between green and roasted beans. Chemometric modeling of the intact peptide profiles delivered excellent classification accuracy between green and roasted coffees (Q^2^ > 0.65 by PLS-DA cross-validation), suggesting a dominant thermal footprint. The identified differential proteins reveal possibly accessible markers for detecting thermal treatment and offer new molecular targets for investigating the mechanisms underlying flavor precursor development.

It should be clear that this study is a proof-of-concept performed with laboratory-scale oven roasting; time–temperature–airflow gradients typical of industrial drum, hot-air, or fluidized-bed roasters were not examined. It should also be noted that while our LC–MS/MS data provide independent evidence that these gene products are expressed in green coffee. However, definitive validation of specific targeted peptides, for instance, by targeted parallel reaction monitoring (PRM) with isotope-labeled peptides, should be required for further studies. Consequently, the differential trends are only valid within the context of the MS study observed, but the biological assignment of the parent proteins remains provisional. The magnitude and kinetics of the observed protein changes should not be extrapolated to commercial roast profiles without further validation. Future work may extend the approach to larger multi-origin sets and correlate marker abundance with sensory data to translate these findings into practical tools for the coffee industry.

## Figures and Tables

**Table 1:** Geographic origin and post-harvest processing method of coffee bean samples used in this study.

Sample ID.	Country of Origin	Region of Produce	Processing Method
1	Colombia	Huila Departamento	Washed
2	Jamaica	N.A. *	Washed
3	Brazil	Minas Gerais State	Natural (Sun-dried)
4	Brazil	Santos	Semi-natural
5	China	Yunnan Province	Natural (Sun-dried)
6	Indonesia	Western Indonesian Time Zone	Washed
7	Ethiopia	Yirgacheffe, Sidama Region	Washed
8	Ethiopia	Yirgacheffe, Sidama Region	Natural (Sun-dried)
9	Ethiopia	Hambella Woreda, Oromia Region	Natural (Sun-dried)
10	Italy (Blended *)	N.A.	Washed
11	China	Yunnan Province	Washed
12	Antigua and Barbuda	Antigua Island	Washed
13	Indonesia	Sulawesi Toraja	Washed
14	Kenya	N.A.	Washed
15	Indonesia	Sumatra Island	Semi-washed
16	Guatemala	Antigua	Washed
17	Papua New Guinea	N.A.	Washed

* Blended, roasters create the “espresso” profile by blending beans from different disclosed regions of production. N.A., not available.

**Table 2:** Total soluble protein of raw and roasted coffee beans. Values are presented as mean ± standard deviation (SD) in grams per 100 g of dry weight (g/100 g DW) for individual sample lots (3 technical replicates per lot).

Sample ID.	Protein Concentration (g/100 g DW)	
	Before Roasting	After Roasting
1	13.36 ± 0.27	4.05 ± 0.19
2	15.00 ± 0.72	4.22 ± 0.16
3	18.08 ± 0.76	4.99 ± 0.45
4	18.03 ± 0.36	5.09 ± 0.49
5	12.11 ± 0.56	3.89 ± 0.43
6	18.48 ± 0.78	9.74 ± 0.55
7	18.78 ± 0.34	10.95 ± 0.19
8	13.61 ± 0.22	10.73 ± 0.25
9	18.04 ± 0.02	9.08 ± 0.50
10	16.21 ± 0.73	4.73 ± 0.46
11	23.33 ± 0.45	6.30 ± 0.49
12	13.08 ± 0.24	9.57 ± 0.29
13	14.21 ± 0.07	3.51 ± 0.47
14	14.03 ± 0.29	5.88 ± 0.32
15	17.99 ± 0.45	10.67 ± 0.49
16	17.19 ± 0.62	10.42 ± 0.43
17	14.34 ± 0.66	10.04 ± 0.47

## Data Availability

The original contributions presented in the study are included in the article/Supplementary Materials; the raw LC-MS files were deposited at https://doi.org/10.25345/C52N4ZX6S; further inquiriescan be directed to the corresponding author.

## Supplementary Materials

The following supporting information can be downloaded at: https://www.mdpi.com/article/10.3390/foods15030538/s1, Table S1. Additional sample information list: Table S2. Data table for chemometrics modeling. Figure S1. Sample images for unroasted and roasted coffee beans. Figure S2. PCA scores plot with sample label. Figure S3. PLS-DA scores plot with sample label.
