# Supplementary material for: Roast-Driven Coffee Proteome Changes Characterized by Bradford Assay, SDS-PAGE, and LC-MS
Source: Foods. 2026 Feb 3;15(3):538. doi: 10.3390/foods15030538 (PMC12896866; doi:10.3390/foods15030538)

Supplementary Material

Roast-Driven Coffee Proteome Changes Characterized by Bradford Assay, SDS-PAGE, and LC-MS

Weiying Lu [a], Yumei Chen [a], Yuge Niu [a*], Liangli (Lucy) Yu [b]

a Institute of Food and Nutraceutical Science, Department of Food Science and Technology, School of Agriculture and Biology, Shanghai Jiao Tong University, Shanghai 200240, China

b Department of Nutrition and Food Science, University of Maryland, College Park, Maryland 20742, United States

* Corresponding Author. E-Mail: yugeniu@sjtu.edu.cn

Table of Contents

Table S1. Additional sample information list

Table S2. Data table for chemometrics modelling

Figure S1. Sample images for unroasted and roasted coffee beans.

Figure S2. PCA scores plot with sample label. The raw and roasted samples were shown in red and green circles, respectively. Each circle represents an independent LC-MS run and is labelled with the corresponding sample ID listed in Table S2. The 95% confidence regions were displayed in ellipses.

Figure S3. PLS-DA scores plot with sample label. The raw and roasted samples were shown in red and green circles, respectively. Each circle represents an independent LC-MS run and is labelled with the corresponding sample ID listed in Table S2. The 95% confidence regions were displayed in ellipses.

Table S1. Additional sample information list

Sample ID.	Complete Product Label (English Translation)	Unit Price*
1	Colombia Huila Supremo Green Coffee Beans - Imported Huila Supremo Raw Coffee Beans	48
2	Jamaica Blue Mountain Green Coffee Beans - Clifton Estate Typica Raw Coffee Beans from Jamaica	105
3	Brazil Minas Anaerobic Natural Green Coffee Beans NY2 FC - Imported Raw Coffee Beans	48.8
4	Brazil Santos Green Coffee Beans - Imported Brazil Santos NY2 Raw Coffee Beans	36.8
5	Yunnan Double Anaerobic Fermentation Natural Green Coffee Beans - Yunnan Small-Bean Raw Coffee	45.8
6	Indonesia WIB Robusta Green Coffee Beans - Imported Indonesian Raw Coffee Beans	28.9
7	Yirgacheffe G2 Green Coffee Beans - Imported Ethiopian Yirgacheffe Raw Coffee Beans	42.8
8	Yirgacheffe Worka Anaerobic Natural Green Coffee Beans G1 - Imported Ethiopian Raw Coffee Beans	95
9	Hambella Natural Green Coffee Beans - Yirgacheffe G1 Specialty Raw Beans, Ethiopia Hambella 9.0 Natural	75
10	Italian Blend Green Coffee Beans - Arabica Blend for Italian Espresso Raw Coffee Beans	55
11	Yunnan AA Green Coffee Beans - Yunnan Small-Bean Raw Coffee Beans, Washed	32.8
12	Guatemala Antigua SHB Green Coffee Beans - Imported Raw Coffee Beans	49.8
13	Indonesia Sulawesi Toraja Imported Green Coffee Beans - Mandheling-style Beans	52
14	Kenya AA++ Green Coffee Beans - Imported Raw Coffee Beans	75
15	Golden Mandheling Green Coffee Beans - Indonesian Sumatra Raw Coffee Beans	79
16	Specialty La Minita La Flor SHB Green Coffee Beans - Imported Guatemalan Raw Coffee Beans	59.8
17	Papua New Guinea AA Green Coffee Beans - Imported Raw Coffee Beans	62.8

*The currency unit is Chinese Yuan (CNY).

Table S2. Data table for chemometrics modelling

ID *	Type	G1	G2	G3	G4	G5	G6	G7	G8	G9
a1-1	Raw	30345.15519	827.1605642	2208.100418	2975.868204	5572.417242	1291.357883	598.5509603	1063.116582	14799.78122
a2-1	Raw	38353.27066	809.8146514	2111.29069	2274.813135	4652.28869	1361.622374	780.2881756	972.9256727	18705.45763
a3-1	Raw	31289.1611	529.2022406	1762.049345	2193.419365	4583.345866	1097.294043	918.1442319	834.9718981	15260.18687
a4-1	Raw	37501.74077	492.438828	1641.585187	1200.692464	3452.315923	1118.500946	898.1047426	805.6321185	18290.1539
a5-1	Raw	40226.58727	646.1435685	1401.54575	2327.826133	3731.508369	1164.271301	866.2509138	938.2255415	19619.10186
a6-1	Raw	45447.14398	734.7596323	3222.49753	6287.447124	5164.936224	669.5883989	994.8465571	561.4449446	22165.24461
a7-1	Raw	35827.53976	342.0505486	1982.740704	1954.647653	3776.871567	727.4535985	537.7418041	815.5374353	17473.62129
a8-1	Raw	35381.43278	287.4450879	1615.809876	1752.624439	4089.914938	579.2314152	496.0466642	708.4143946	17256.04832
a9-1	Raw	29492.45927	245.7797449	1620.190405	1454.790214	4349.66208	497.2892758	410.1569644	786.8499709	14383.90879
a10-1	Raw	33178.31668	488.2570403	2180.250344	2329.92107	5725.875329	782.2030371	1074.073242	743.9279516	16181.556
a11-1	Raw	41776.60118	818.5568288	2376.452001	2075.979212	5130.197537	1329.109027	1038.258898	940.1769988	20375.06658
a12-1	Raw	42410.49971	918.9198446	2212.575153	2129.811732	5104.314311	1187.293232	859.7283473	1154.241122	20684.22827
a13-1	Raw	32052.76355	599.4735988	1892.954755	3608.856846	4780.394755	1070.538676	818.5310327	823.5256559	15632.60708
a14-1	Raw	28577.66109	386.0052933	1798.493532	2483.238312	5390.947784	803.3604815	828.8238468	642.5380689	13937.74819
a15-1	Raw	30999.68851	483.4865327	2093.920327	2687.095442	5321.159282	849.0332254	712.9351025	730.6594056	15119.0068
a16-1	Raw	39519.55961	868.1129517	2233.032697	2427.443887	5271.17383	1201.043313	747.8078069	995.9289824	19274.27401
a17-1	Raw	38043.12301	432.8634782	2209.291905	2341.081771	5205.792053	945.691786	620.4452061	804.6431923	18554.19403
a1-2	Raw	46125.36008	262.7296695	1938.314895	1493.286726	5784.595854	772.6374591	662.1212635	541.1816189	22496.02064
a2-2	Raw	57280.95807	289.4542702	1975.262405	912.87838	4641.003265	807.0701681	829.2472082	390.5766567	27936.77086
a3-2	Raw	38356.83598	134.5552567	977.4524191	443.4000566	3335.169503	399.407468	729.0504579	223.4725933	18707.19649
a4-2	Raw	48493.42668	173.3071656	999.4586348	195.5679948	2951.819651	472.7159085	849.3825472	226.4244711	23650.96177
a5-2	Raw	59578.36024	157.4393134	969.381182	912.7897212	3504.198268	675.5522419	804.7267578	334.6994365	29057.24789
a6-2	Raw	28451.40352	3.559928983	1007.81345	1002.548248	1704.301304	5.161647099	286.4146228	0	13876.1705

a7-2	Raw	34554.91873	64.51772964	745.6988473	237.983621	2116.297882	93.54617849	254.9142946	178.0244148	16852.94519
a8-2	Raw	31431.24263	53.58750355	453.3747603	184.3378922	1938.410451	65.15379459	138.5253343	103.4390686	15329.48213
a9-2	Raw	28284.13539	73.93212194	681.8751831	205.232911	2493.265075	98.04957191	146.0543829	137.3108737	13794.59136
a10-2	Raw	42779.45329	113.7692907	1483.609586	779.195518	4723.958216	346.1612775	902.9514442	131.7512126	20864.17239
a11-2	Raw	54871.39168	266.2333216	1745.338848	648.7688306	3673.606846	745.1421107	783.6561778	290.6386165	26761.58967
a12-2	Raw	66236.92194	477.5012944	2126.602253	1116.587178	4876.792752	891.2872611	738.287831	651.7926574	32304.72696
a13-2	Raw	48644.93339	252.1310962	1571.450472	2292.637887	4853.474023	750.0633042	778.0968921	303.0071981	23724.85383
a14-2	Raw	29741.55537	78.89955126	824.4935038	468.8451275	3123.859194	175.1378628	396.3210164	94.7939164	14505.39665
a15-2	Raw	41968.37822	156.0218718	1412.760285	1082.166401	4746.273612	385.8997267	477.7578895	154.3535415	20468.59908
a16-2	Raw	61552.49818	320.3854231	2149.72542	1357.281873	5496.549575	838.1674457	613.2186484	485.4438473	30020.06418
a17-2	Raw	51346.39373	109.5879639	1540.257201	988.2102241	4567.333992	369.5637181	386.2176941	165.5474021	25042.39601
r1-1	Roasted	25808.28083	639.1035575	262.1736815	633.9375412	1895.861281	964.8765611	189.1195239	474.8290583	12587.08046
r2-1	Roasted	76307.55835	1659.204712	381.1171325	84.16585529	385.9292708	1473.646008	840.5099902	1863.168481	37216.32536
r3-1	Roasted	29852.83395	969.9264374	32.50906768	52.68142619	63.74665881	812.7767854	455.5269097	1290.088921	14559.66886
r4-1	Roasted	53479.45254	2152.609778	219.7078436	53.27393385	61.02122613	1357.03668	912.9775261	1916.378908	26082.72036
r5-1	Roasted	60370.83805	1857.200687	94.03594956	47.47939823	128.5068806	1523.669375	803.5611626	1626.800811	29443.75104
r6-1	Roasted	62707.31299	1756.209794	163.4126358	42.17686884	92.30840894	372.4047273	509.2244857	1739.118954	30583.28445
r7-1	Roasted	76563.54091	1948.959211	1136.782575	315.2806856	1703.204822	1700.129643	883.5468262	1662.014466	37341.17184
r8-1	Roasted	90249.02545	1855.2095	664.4091559	329.0904688	1206.68432	1625.905212	918.2618938	1896.452714	44015.78516
r9-1	Roasted	86194.73621	1599.001721	470.5500033	129.7446584	644.6310394	1590.971345	653.6262471	2057.271098	42038.4483
r10-1	Roasted	43651.34882	1509.909798	20.40478878	61.38978714	98.05823671	1387.036044	395.0541251	1847.479074	21289.40875
r11-1	Roasted	97731.705	3272.798486	547.6014313	97.57818843	949.9978149	2554.105039	1543.940828	2058.262229	47665.1987
r12-1	Roasted	91420.02053	2881.546902	735.5189794	215.0258523	1520.423601	2545.899323	1481.912225	2184.307676	44586.89679
r13-1	Roasted	18554.99166	760.9445632	23.80544088	18.8188197	133.3015174	1302.08963	119.5418749	651.1108841	9049.543997
r14-1	Roasted	310.278344	0	0	0	0	0	6.740750023	0	151.3273396
r15-1	Roasted	70542.91043	880.4652974	65.76702717	15.99020229	112.3461122	655.8344175	492.1171448	1192.765199	34404.82127

r16-1	Roasted	78953.44942	1307.444843	41.8375971	7.977992875	238.7268187	956.7590215	727.7818798	1540.667038	38506.76559
r17-1	Roasted	83690.53261	1075.695014	120.4182217	34.32309613	670.6715762	957.1761791	619.2000161	1367.031523	40817.11115
r1-2	Roasted	6802.998917	0	0	0	57.9288298	29.26448394	0	99.63934214	3317.923238
r2-2	Roasted	55623.12865	554.0811006	33.46440802	0	32.70814823	417.0735336	282.016898	1194.62933	27128.22292
r3-2	Roasted	13659.96349	121.6694052	0	0	0	43.33102156	70.15098849	559.182644	6662.166325
r4-2	Roasted	29892.97138	544.623033	40.87669951	0	0.580854081	159.0360786	193.9007934	865.5487163	14579.24448
r5-2	Roasted	38172.75464	461.4061674	0	0	0	298.4034007	156.1723687	974.6711619	18617.41729
r6-2	Roasted	36013.10209	351.992718	7.213628632	0	1.687378449	4.41049914	93.45389146	967.1401085	17564.12279
r7-2	Roasted	71165.79564	744.3067576	275.5308756	18.11533347	782.2953272	706.5500499	389.0348734	1056.380955	34708.6116
r8-2	Roasted	77262.27799	756.0127042	98.14593883	42.88843454	409.1053128	626.2852621	415.9598853	1280.599305	37681.95625
r9-2	Roasted	63466.77685	464.0627393	30.61560875	10.77737562	81.36346636	485.297183	174.6005457	1198.732444	30953.68621
r10-2	Roasted	19948.98127	311.704065	0	0	0	132.3168062	13.27587936	594.0093015	9729.413356
r11-2	Roasted	69956.04488	1400.573465	35.98403239	0	98.13742014	967.4085484	694.0769382	1267.428763	34118.59825
r12-2	Roasted	69371.49927	1235.31145	101.9524426	8.646811974	422.1473229	942.3712955	664.9970696	1349.919994	33833.50671
r13-2	Roasted	3101.044214	22.33409448	0	0	0	111.1848517	0	62.99929309	1512.425151
r14-2	Roasted	388.4548297	0	0	0	0	0	4.804449297	0	189.4551685
r15-2	Roasted	67640.04272	747.008432	33.70948586	0	88.97007915	541.5824247	391.7758079	1187.257132	32989.04973
r16-2	Roasted	74354.11501	1241.359587	27.04240368	8.274881778	156.6619115	802.354338	631.7192891	1510.478948	36263.60214
r17-2	Roasted	76584.19776	879.1908861	109.1773161	22.72582312	585.789715	760.5679504	589.555182	1285.460436	37351.24649

*sample ID is given in the format of (a/r)n_a-n_b, where a/r indicate sample class (raw or roasted bean), n_a is the sample ID in the manuscript and n_b represent different replicates.

Figure S1. Sample images for unroasted (a) and roasted (b) coffee beans.

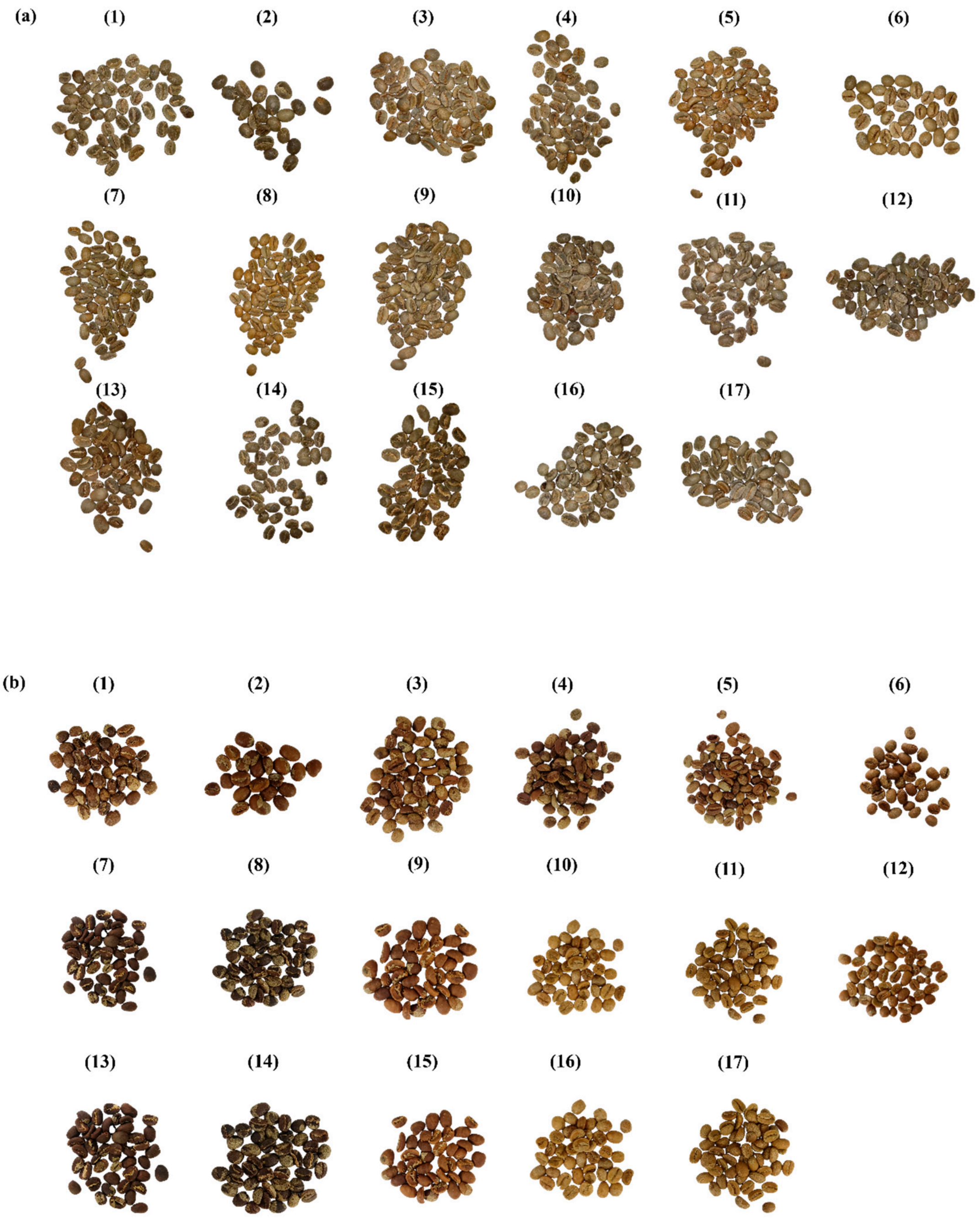

Figure S2. PCA scores plot with sample label. The raw and roasted samples were shown in red and green circles, respectively. Each circle represents an independent LC-MS run and is labelled with the corresponding sample ID listed in Table S2. The 95% confidence regions were displayed in ellipses.

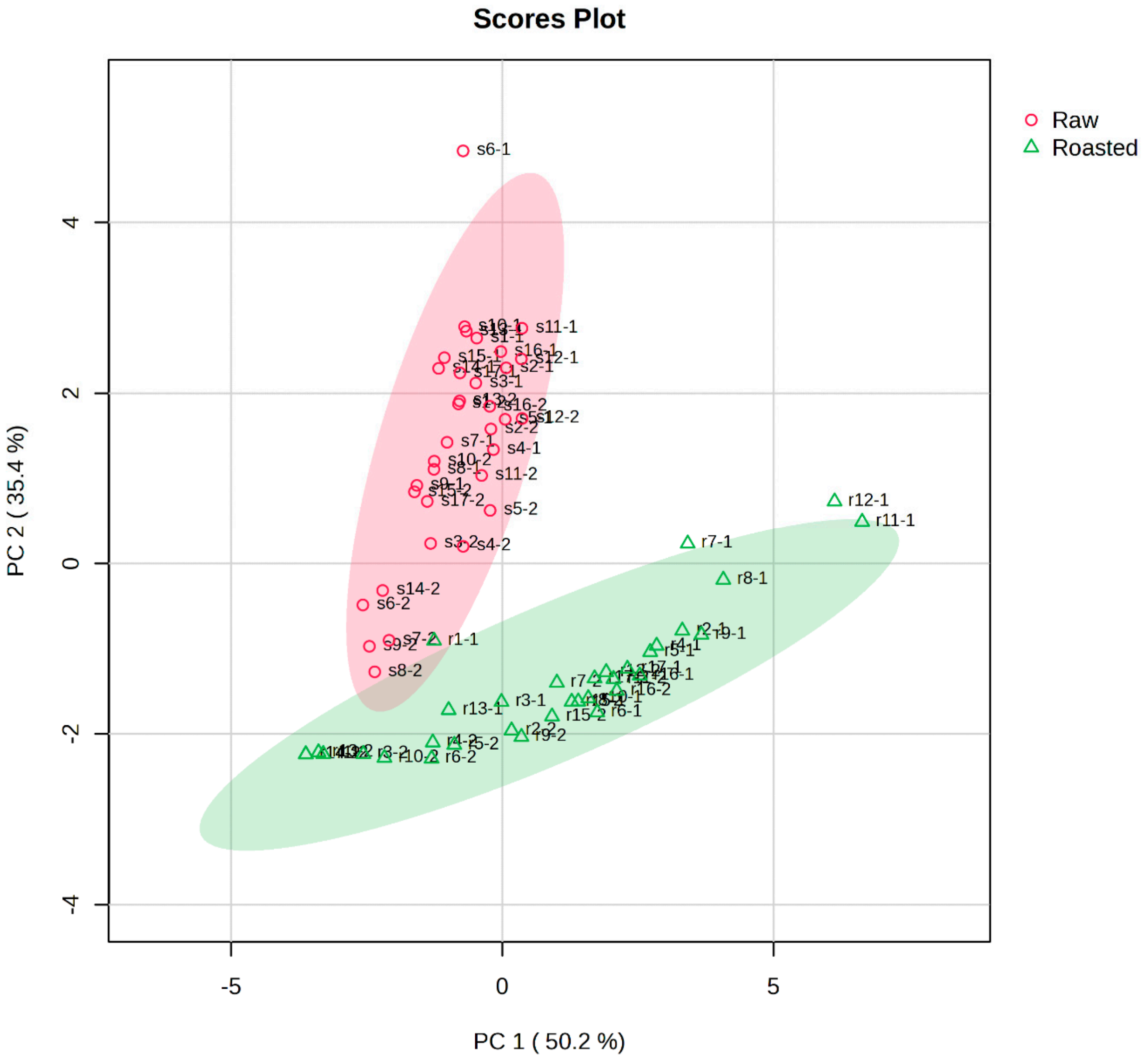

Figure S3. PLS-DA scores plot with sample label. The raw and roasted samples were shown in red and green circles, respectively. Each circle represents an independent LC-MS run and is labelled with the corresponding sample ID listed in Table S2. The 95% confidence regions were displayed in ellipses.

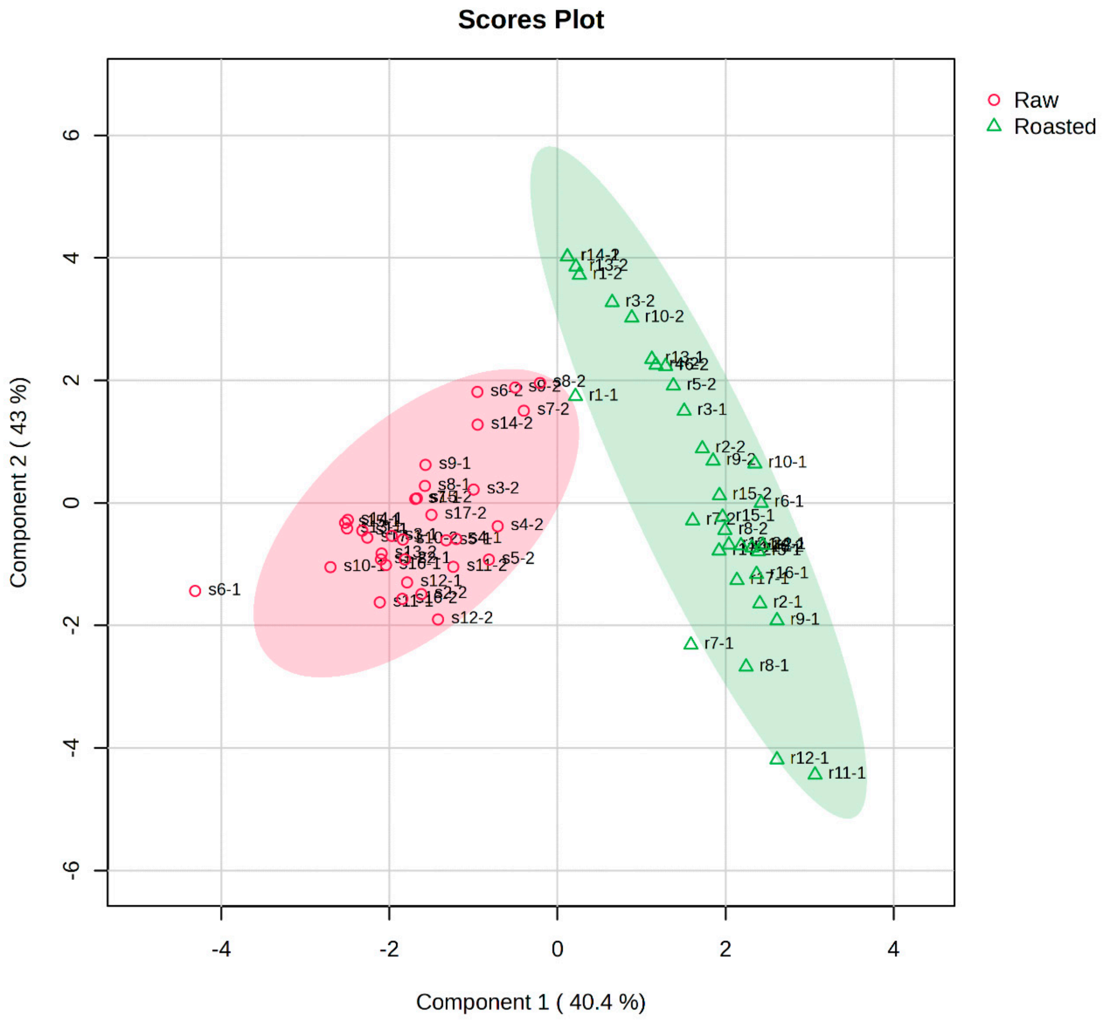

Supplement: Supplementary file 1 [file foods-15-00538-s001.zip › foods-4090222-supplementary.pdf]
